# Cocowood Fibrovascular Tissue System—Another Wonder of Plant Evolution

**DOI:** 10.3389/fpls.2016.01141

**Published:** 2016-08-09

**Authors:** Oswaldo M. González, Khoi A. Nguyen

**Affiliations:** ^1^Research Department, Universidad de las Fuerzas Armadas ESPESangolqui, Ecuador; ^2^Griffith School of Sciences and Engineering, Griffith UniversityGold Coast, QLD, Australia

**Keywords:** coconut palm stem tissue (cocowood), monocotyledon plant, fibrovascular bundle degree of orientation, mechanical efficiency, finite element analysis, integral and macroscopic levels of hierarchical structure

## Abstract

The coconut palm (*Cocos nucifera L*.) stem tissue (referred to as *cocowood* in this study) is a complex fibrovascular system that is made up of fibrovascular bundles embedded into a parenchymatous ground tissue. The complex configuration of fibrovascular bundles along with the non-uniform distribution of the material properties likely allow senile coconut stems to optimize their biomechanical performance per unit mass (i.e., mechanical efficiency) and grow into tall, slender, and very flexible plants with minimum resources of biomass and water. For the first time, to the best of the authors' knowledge, this paper examines, from the integral (i.e., stem structure) and macroscopic (i.e., tissue structure) levels of hierarchy, the characteristic triple helix formation depicted by the fibrovascular bundles within the monocotyledon cocowood. The natural course of the tangential orientation of the axial fibrovascular bundles is mapped for the whole cocowood structure by quantifying 264 cocowood discs, corresponding to 41 senile coconut palms estimated to be >70 years old. The observed variations were modeled in this paper by simple equations that partially enabled characterization of the cocowood fibrovascular tissue system. Furthermore, 11 finite element analyses (FEA) were performed over a three dimensional (3D) finite element (FE) model resembling a characteristic coconut palm stem of 25 m in height to analyze the biomaterial reactions produced by the progressive deviation of the tangential fibrovascular bundles on the cocowood mechanical response (i.e., on the material compressive strength and the bending stiffness). The analyses in this study were carried out for the critical wind speed of 23 m/s (i.e., *Gale* tornado according to the Fujita tornado scale). For each analysis, the characteristic average maxima degree of orientation of the cocowood fibrovascular bundles was varied from 0° to 51°. The acquired results provided a deep understanding of the cocowood optimum fibrovascular tissue system that denotes the natural evolution of the material through millions of years. The knowledge advanced from this study may also serve as concept generators for innovative biomimetic applications to improve current engineered wood products.

## Introduction

### Cocowood fibrovascular tissue system

**Figure 1:**
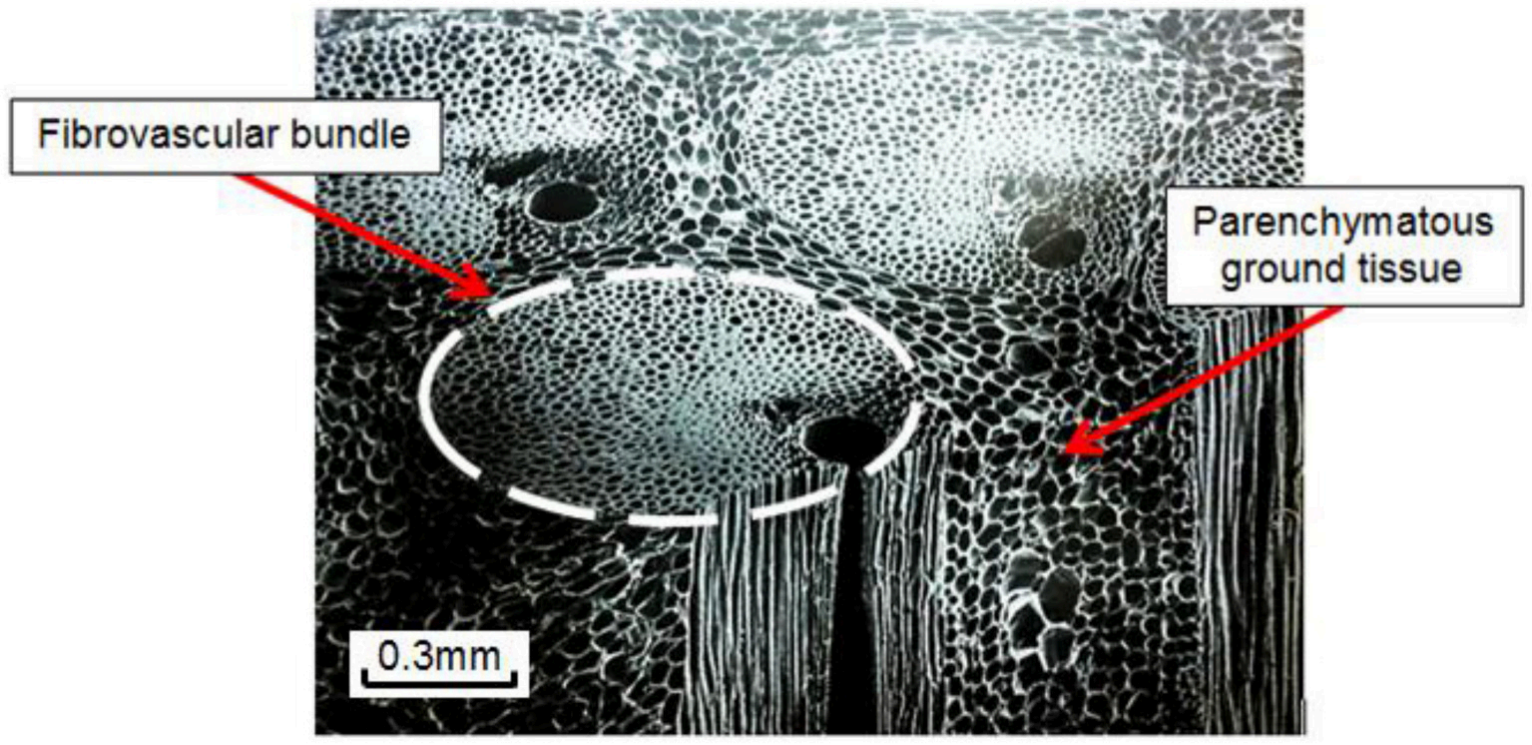
**Cocowood is a complex vascular tissue system that depicts a cellular structure that can be described as parallel stiff fibers embedded in foam**. Its simple botanical description is a blend of two structurally and functionally divergent cell tissues: (a) the fibrovascular bundles with a honeycomb-like structure, which are rounded and dense groups of long fibers surrounding vessel elements for sap circulation, mainly orientated in the longitudinal axis of the stem, and (b) the ground tissue made up of foam-like polyhedral parenchyma cells.

According to botanical taxonomy, coconut palms (*Cocos nucifera L*.) belong to the palm tree family (*Arecaceae*), a family of monocotyledon plants whereas hardwoods and softwoods are woody plants with similar stem characteristics from the point of view of structural mechanics, but different tissue properties (e.g., quasi-uniform vs. non-uniform density distribution; Bahtiar et al., 2010). The main differences between palmwoods, hardwoods, and softwoods are summarized by Butterfield et al. (1997). The coconut palm stem is composed of two anatomical components: the cortex and the central cylinder. The cortex is a fibrous tissue covering the entire outer circumference of the stem. It is typically 1–1.5 cm thick and plays a similar function to that of the bark in woody plants (Tomlinson, 1990). The central cylinder is a plant tissue (referred to as cocowood herein) that constitutes more than 96% of the palm stem. Cocowood is a complex tissue that depicts a cellular structure (Gibson, 2005) comprising two different tissues as shown in Figure 1.

The fibrovascular tissue system (i.e., the assemblage of conducting tissues and associated supportive fibers) of monocotyledonous palms has been extensively investigated from different levels of hierarchy (Zimmermann and Tomlinson, 1972; Tomlinson et al., 1984, 2001, 2011; Tomlinson, 1990, 2006; Rüggeberg et al., 2008, 2009; Thomas and De Franceschi, 2013). The system is essentially made up of three elements: axial fibrovascular bundles, leaf traces, and fibrous cortical traces (Zimmermann and Tomlinson, 1972). The axial fibrovascular bundles remain within the stem tissue and provide longitudinal continuity to the palm structure (Tomlinson, 1990). Both leaf and cortical traces connect the stem fibrovascular system with that of the leaves and petioles (Rüggeberg et al., 2009). Moreover, the leaf traces are connected to the axial fibrovascular bundles within the stem central cylinder by narrow bundles called bridges (Tomlinson, 1990).

The fibrovascular bundles are made of fibers, xylem, phloem, and axial parenchyma. The fibers constitute over half of the volume of each fibrovascular bundle (Butterfield et al., 1997). In monocotyledonous palms, the fibers have thin walls and large lumens (cavities inside the fibers) toward the stem core, and thick walls and small highly lignified lumens toward the stem periphery (Fathi et al., 2014). This implies denser and stronger fibers at the stem periphery likely influencing the biomechanical performance of palm stems, in terms of bending strength (Sudo, 1980; Rich, 1986, 1987; Butterfield et al., 1997; Fathi and Frühwald, 2014) and bending stiffness (Rüggeberg et al., 2009). The fiber walls are commonly multi-layered (e.g., similar to cell wall layers of hardwood fibers) and the thickness and microfibril orientation of each layer are clearly visible under light microscopy (Butterfield et al., 1997).

Thousands of fibrovascular bundles (e.g., 20,000 approximately) are scattered within a single transverse section of a senile (i.e., <60 years old) coconut palm stem (Zimmermann and Tomlinson, 1972; Tomlinson, 1990). Radial density and fibrovascular bundle distribution develop non-homogeneously throughout the palm stem, with denser tissue toward the periphery and base, where the biomechanical performance of the material shows maximum bending stresses (Rüggeberg et al., 2009; Gibson, 2012). According to Killmann and Fink (1996), the distribution of fibrovascular bundles within coconut stems can be classified into three different zones: (a) the dermal zone, close to the cortex, with a high density distribution of about 68 fibrovascular bundles per cm^2^, (b) the sub-dermal zone, with a medium density distribution of about 42 fibrovascular bundles per cm^2^, and (c) the central zone, with a low density distribution of about 18 fibrovascular bundles per cm^2^. The average cross-cut area of the single fibrovascular bundle within cocowood stems was found to vary from 0.30 mm^2^ at the central zone to 0.53 mm^2^ at the dermal zone (Fathi et al., 2014). The high and low distributions of fibrovascular bundles toward the palm stem periphery (dermal zone) and core (central zone), respectively, reflect an efficient structure that is not excessively overbuilt (Tomlinson, 1990).

### Fibrovascular bundle degree of orientation in palmwoods

In plant species, fibers can be oriented in various directions within the structure to adapt the structural response to the external loading conditions (Zhang et al., 2012). The fibrovascular development (i.e., the method of construction of the fibrovascular tissue system described in Section Cocowood Fibrovascular Tissue System) of monocotyledon palms was thoroughly analyzed by Tomlinson (1990), especially for that of *Rhapis excelsa* palms. The course of the fibrovascular bundles was found to follow a helical path as they ascend the stem, but also vary progressively toward the center of the stem before bending rapidly outwards toward a leaf trace at the periphery of the stem. While the observed pattern is typical, it is not a consistent attribute for all palm species. According to Tomlinson (1990), the longitudinal helical pattern seems to have been observed in the nineteenth century by both Meneghini (1836) and De Mirbel (1843). Tomlinson (1990) pointed out that the helical formation “*probably adds to the mechanical efficiency of the stem by minimizing longitudinal splitting*.”

The natural course of axial fibrovascular bundles within monocot palm stems was further described by Rüggeberg et al. (2008) as a feature that follows both a “*screw-like pathway*” (i.e., a longitudinal—helical course) while running up the stem, and a “*zigzag*” pattern across the stem diameter, thereby deviating in tangential and radial directions as they run back and forth within the palm stem. Butterfield et al. (1997) called attention to “*an understanding of the three dimensional path-ways traced out by the fibrovascular bundles within the stem that is fundamental to a full understanding of the structure of palmwood*.”

Rüggeberg et al. (2009) by studying the structure–function relationships of different vascular bundle types in one 33 year-old *Washingtonia robusta* palm at an elevation of 5 m graphically reported the radial degree of orientation of the fibrovascular bundles. The bundles were found to move toward the periphery of the stem, with the higher degree of orientation reported in the dermal zone of the stem.

From the literature review, it appears that, only one study performed by Kuo-Huang et al. (2004) has reported a partial degree of orientation of axial fibrovascular bundles within juvenile coconut stems (20–25 years old), average heights of 6 m. Kuo-Huang et al. (2004) graphically showed a tangential degree of orientation of fibrovascular bundles across the stem diameter that varied from 4° to 12° at the bottom of the studied stems.

**Figure 2:**
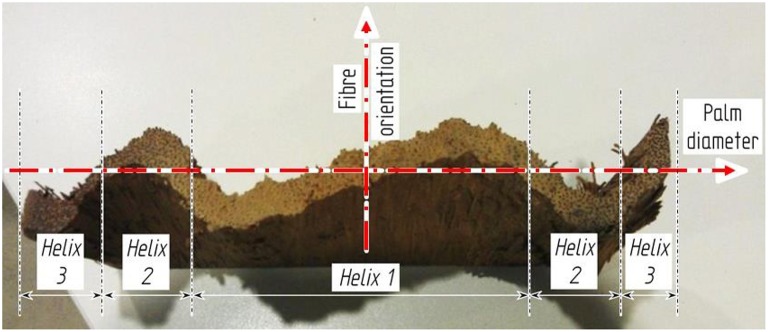
**Cocowood triple helix pattern shown in a radially split disc**. Similar to the *zigzag* pattern described by Rüggeberg et al. (2008) for *Washingtonia robusta* palms, the cocowood fibrovascular bundle architecture follows an axisymmetric and triple helix configurations across the palm stem diameter.

Although the development of the fibrovascular system of monocotyledonous tissues has been investigated at different hierarchical levels, and the triple helix formation of cocowood fibrovascular bundles (Figure 2) has been observed, they have not yet been characterized and modeled for the whole cocowood structure. Furthermore, the extent to which the degrees of orientation of the fibrovascular bundles influence on the cocowood stem biological function has remained unknown up to now. These observations were the driving forces behind the work in this study.

## Materials and methods

### Tangential fibrovascular bundle degree of orientation

**Figure 3:**
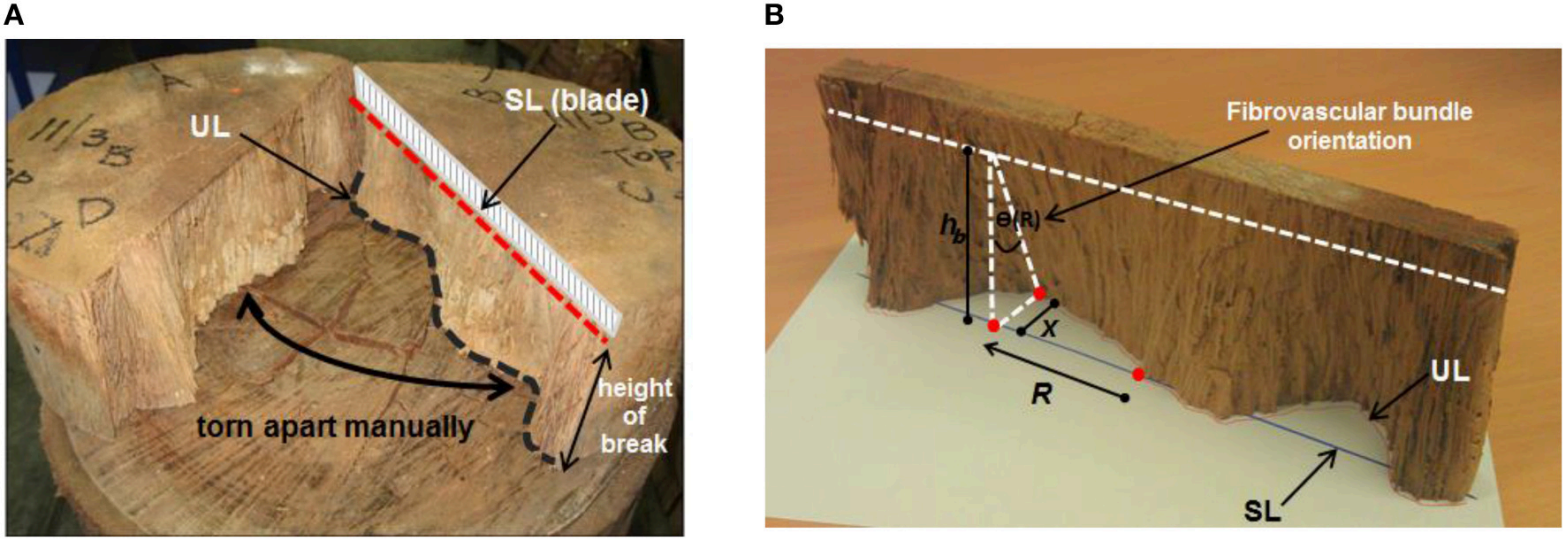
**(A)** Each disc was split up to a depth of 10 mm with a 1100 mm long lathe-blade and manually torn apart. This left a straight split line (SL) on the upper face of the disc, where the blade was driven, and an undulated split line (UL) on the lower face that followed the fibrovascular bundles. **(B)** Measurement of the tangential fibrovascular bundle degree of orientation.

A total of 41 senile coconut palms with average stem heights ranging from 18.8 to 25 m, sourced during the Project FST/2004/054: “Improving value and marketability of coconut wood” by the Australian Centre for International Agricultural Research (ACIAR) in 2010, from the Pacific islands of Fiji (29 palms) and Samoa (12 palms), were used to measure the tangential orientation of the cocowood axial fibrovascular bundles. For each palm, nominal 50 mm thick discs were cut at the key stem elevations of 0.2, 3.2, 6.2, 9.4, 12.4, 15.8, 18.8, 22.0, and 25.0 m. The fibrovascular bundle degrees of orientation were acquired by measuring the tangential deviation of the fibrovascular bundles after splitting the discs along their diameter (Figure 3A). One of the two split half-disc was positioned on a white paper sheet to project the two lines in the same plane (i.e., the straight split line and the undulated split line). The straight line (SL) was projected perpendicularly onto the paper and the undulated line (UL) was directly drawn on the paper sheet. Each line was divided into 100 points equally spaced along the disc diameter to take measurements. The tangential fibrovascular bundle degree of orientation θ at a radial position *R* from the disc center was calculated for each point as,

(1) $$\theta \left(R\right)=\;tan^{-1}\left(\frac{x}{h_{b}}\right)$$

where *x* is the distance between the lines UL and SL, and *h*_*b*_ is the measured disc height of break, as shown in Figure 3B.

As mentioned before, the tangential fibrovascular bundle degree of orientation of the coconut stem tissue describes a triple helix structure. Observations from the collected data showed that the fibrovascular bundle degree of orientation is typically not vertical at the periphery of the palm. As the distribution of the fibrovascular bundle degree of orientation is axisymmetric (i.e., symmetrical about the palm stem's main axis), the fibrovascular bundles at the center of the stem must be vertical when considered analytically. The following periodic equation is proposed to characterize the fibrovascular bundle degree of orientation θ as function of the radial position *R* from the center of the stem and the palm height *h*,

(2) $$\theta \left(R,h\right)=\theta_{max}(h)\times sin\left(2\pi \times f\times \frac{R}{R_{max}\left(h\right)}\right)$$

where *R*_*max*_ is the palm maximum radius, *f* is the natural frequency of the triple helix, and θ_*max*_ is the average maxima tangential fibrovascular bundle degree of orientation. Average measured maximum radiuses of the studied cocowood discs (see Table 1) allowed determining the characteristic palm radius *R*_*max*_ (in mm) in terms of the palm stem height *h* (in m) as,

(3) $$R_{max}\left(h\right)=\frac{4035}{h+\text{25}.\text{5}}$$

**Table 1:** **Measured maximum radiuses at the studied key stem elevations**.

**Palm stem height (m)**	**Number of measurements (discs)**	**Average radius (mm)**	***CoV***
0.2	34	164.88	0.14
3.2	32	134.14	0.08
6.2	30	124.04	0.11
9.4	30	115.92	0.10
12.4	30	107.43	0.11
15.8	28	96.63	0.12
18.8	28	90.35	0.15
22.0	26	87.78	0.11
25.0	26	79.11	0.16

**Figure 4:**
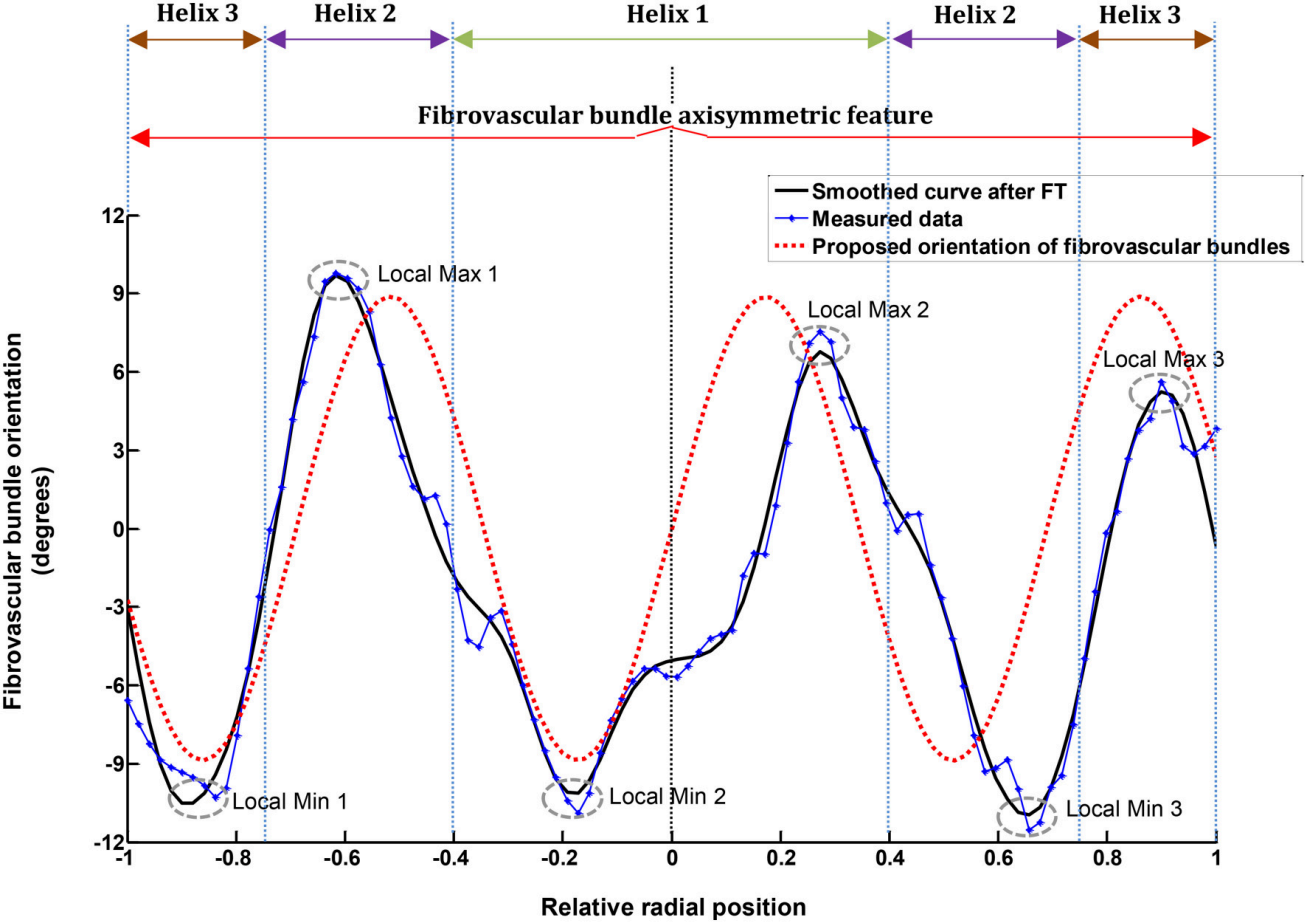
**Measured, attenuated (after applying FT), and proposed tangential fibrovascular bundle degree of orientation for palm 13 (Fiji) at 12.4 m high**. The characteristic cocowood fibrovascular bundle degree of orientation proposed herein (i.e., the red segmented curve) provides similar results to the actual measurements for the given palm; it also shows the axisymmetric and triple helix configurations of the fibrovascular bundles.

**Figure 5:**
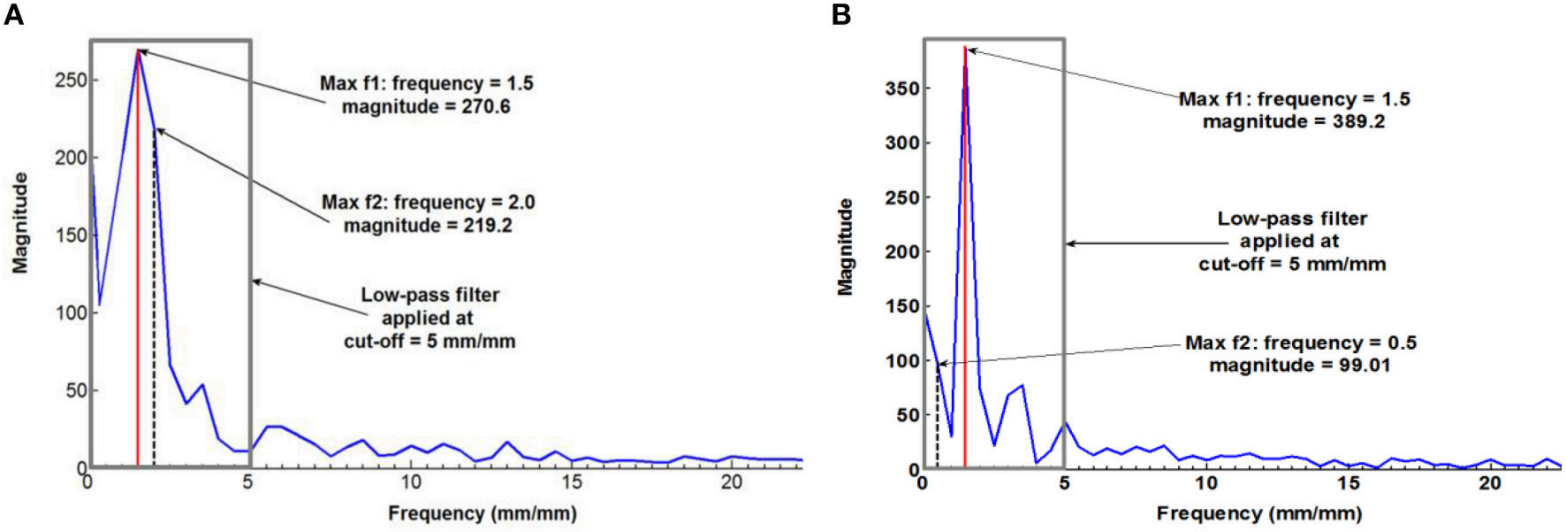
**Frequency spectrum with (A) no clear peak [palm 15 (Fiji) at 0.2 m high], and (B) one dominant frequency [palm 21 (Fiji) at 0.2 m high]**.

Figure 4 plots a typical measured fibrovascular bundle degree of orientation θ across the palm stem diameter, showing the axisymmetric distribution and the triple helix pattern for the palm 13 at 12.4 m of stem height. To suppress the noise inherent to the fibrovascular bundle degree of orientation measurements, a discrete Fourier transform (FT) was applied to all measurements. The FT function allows transforming the discrete fibrovascular bundle degree of orientation signal from the length domain to the frequency domain. It expresses the signal as a sum of sinusoids of different frequency and magnitude (Winter David, 1997). To smooth the curves resulting from the raw measurements of fibrovascular bundle degrees of orientation, the high frequency noise was removed by applying a low-pass filter to the transformed signal, with a cutoff frequency of 5 mm/mm (Figure 5). The inverse Fourier transform was subsequently applied to obtain the smoothed fibrovascular bundle degree of orientation curve. A typical smoothed fibrovascular bundle degree of orientation curve is shown above in Figure 4. The representative average maxima tangential fibrovascular bundle degree of orientation θ_*max*_ was then calculated for each cocowood disc on the smoothed curve (see Figure 4) as the arithmetic mean value of the three local minimum and the three local maximum fibrovascular bundle degrees of orientation,

(4) $$\theta_{max}=\frac{1}{6}\left(\sum_{i\;=\;1}^{3}\theta_{max,i}+\sum_{i\;=\;1}^{3}\left|\theta_{min,i}\right|\right)$$

where θ_*min, i*_ and θ_*max, i*_ are the values of the *i*th local minimum and maximum, respectively. The natural frequency *f* of the triple helix was calculated from the frequency spectrum of the discrete FT applied to the tangential fibrovascular bundle degree of orientation measurements of each disc. With the discretization of 100 measurements, two different types of frequency spectra occurred: (i) two distinct dominant frequencies with no clear peak were observed, as shown in Figure 5A, and (ii) only one dominant frequency was observed, as shown in Figure 5B. When no clear peak was observed, the two dominant frequencies were considered to both contribute to the signal if their magnitudes do not differ by more than 25%. In this case, the triple helix frequency *f* was approximated as the average of these two dominant frequencies. Otherwise, the frequency with the higher magnitude was considered to predominantly contribute to the signal and the helix frequency *f* was taken as this frequency.

### Finite element modeling

**Figure 6:**
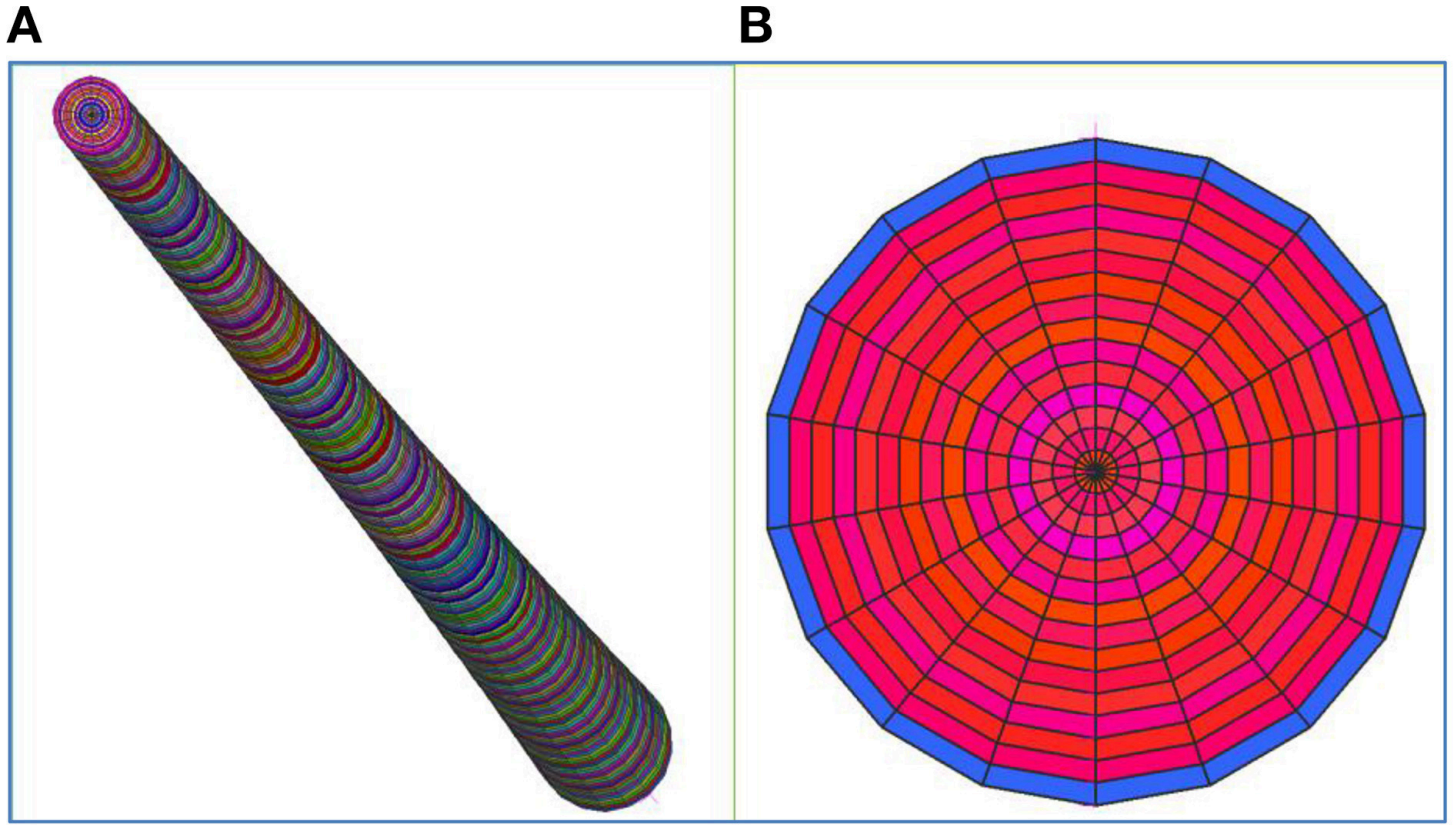
**A 64,800 brick element FE model, with a maximum aspect ratio of about 10, was selected as optimum for carrying out the finite element analyses**. The cocowood optimum FE model included 240, 18, and 15 divisions, axially, peripherally, and radially, respectively. The whole domain in the FE model was meshed with 65,311 nodes. **(A)** Axial perspective of the characteristic coconut stem model, **(B)** Cross-sectional area at the bottom of the model. Note that the color coding in the figure is reflecting brick elements with different properties.

The 3D FE model of a characteristic senile coconut palm stem shown in Figure 6 was built using the commercial finite element software Strand7 (2005). From the structural mechanics point of view, palm stems can be considered as composite, hierarchically structured, and fiber-reinforced beams (Speck and Burgert, 2011). Therefore, the cocowood FE model herein bears a resemblance to a fully fixed (i.e., all the model bottom surface nodes were constrained to the ground) cantilever tapered beam or pole with foliage but no branches. The structural modeled system has consequently one degree of freedom. In the FE model, the characteristic form, complex structure of the coconut stem-tissue and the cocowood properties were all derived from previous findings by the author (Gonzalez et al., 2014, 2015; Gonzalez, 2015). Table 2 gives characteristic green properties [i.e., basic density (*d*_*b*_), modulus of elasticity (MOE), compressive modulus of rupture (MOR), shear modulus (*G*), and shear strength (τ_*max*_)] at key locations of the cocowood FE model, in the longitudinal (L), radial (R), and tangential (T) directions.

**Table 2:** **Green material properties for a characteristic senile coconut palm stem**.

**Stem's key location**	**Radial position**	***d_b_* (kg/m^3^)**	**MOE_L_ (MPa)**	**MOE_R_ (MPa)**	**MOE_T_ (MPa)**	**MOR_L_ (MPa)**	**MOR_R_ (MPa)**	**MOR_T_ (MPa)**	***G_LT_* (MPa)**	***G_TR_* (MPa)**	***G_RL_* (MPa)**	***^τ^max_LT* (MPa)**	***^τ^max_TR* (MPa)**	***^τ^max_RL* (MPa)**
Bottom (0 m)	Periphery	901	8608	463.9	352.3	40.2	16.8	15.3	783.2	154.9	930.3	9.9	2.3	10.2
	Core	223	1313	87.6	88.9	6.0	1.2	0.8	79.4	37.2	161.2	1.0	0.2	1.2
Top (25 m)	Periphery	595	5315	294.1	233.4	24.8	9.8	8.8	465.6	104.6	588.2	5.9	1.1	6.1
	Core	112	118.3	26.0	45.8	0.4	0.0	0.0	0.0	14.8	25.5	0.0	0.0	0.0

The orthotropic material behavior and the radial triple helix configuration depicted by the tangential deviation of the axial fibrovascular bundles were captured in the cocowood FE model by using the 3D brick orthotropic element property from Strand7 (i.e., 8-node brick elements have different properties for the *L, R*, and *T* directions, depending on their location within the FE model). The number of brick elements along the radius, height, and perimeter of the cocowood FE model, was tested until reaching the most appropriate mesh for the analyses. Thus, a regular solid mesh was generated to uniformly distribute the nodes in the FE model that allowed a uniform application of the wind pressure profile. Besides, the stresses developed within the cocowood model by the applied loading conditions described below, were calculated by Strand7 at the centroid of each brick element (i.e., the resultant brick stress value reflected the average of the corresponding eight brick Gauss points). Specifically, eight finite element models were analyzed at the acute wind speed of 60 m/s (i.e., Significant tornado according to the Fujita tornado scale; Cullen, 2002) to define the most appropriate FE model for the current analyses. Table 3 gives detailed information about each analyzed model. Two aspects were considered when defining the optimum model in this study: (i) an aspect ratio no >10 for each brick element, and (ii) a time no >1 h 15 min to process the model. Additionally, three parameters were considered to find the point of convergence when the total number of brick elements was varied in the model: (i) the maximum stress occurring in the axial direction of the model; (ii) the vertical displacement at the top/middle node of the FE model, and (iii) the displacement at the top/middle node of the model in the wind direction.

**Table 3:** **Results of the mesh and convergence studies**.

**FE model**	**Analyzed wind speed**	**Total brick elements**	**Number of divisions in the FE model**			**Brick size**			**Brick aspect ratio**			**Time to process the FE model**	***Max* axial stress**	**Top/middle node displacements**	
**no**.	**(m/s)**		**Radially** **(R)**	**Axially** **(L)**	**Peripherally** **(P)**	**(R)** **(mm)**	**(L)** **(mm)**	**(P)** **(mm)**	**(P/R)**	**(L/R)**	**(L/P)**		**(MPa)**	**Wind direction** **(mm)**	**Vertical direction** **(mm)**
1	60	2100	15	10	14	10.53	2500.00	70.91	6.7	237.3	35.3	11 s	17.08	4473.68	508.59
2	60	4200	15	20	14	10.53	1250.00	70.91	6.7	118.7	17.6	46 s	44.50	11,274.95	3521.02
3	60	8400	15	40	14	10.53	625.00	70.91	6.7	59.3	8.8	3 min 40 s	68.05	15,907.14	8097.57
4	60	21,600	15	80	18	10.53	312.50	55.15	5.2	29.7	5.7	17 min 51 s	77.87	17,232.41	10,330.57
5	60	43,200	15	160	18	10.53	156.25	55.15	5.2	14.8	2.8	44 min 34 s	80.19	17,602.04	10,717.32
6	60	54,000	15	200	18	10.53	125.00	55.15	5.2	11.9	2.3	57 min 51 s	80.45	17,745.97	10,818.48
7^*^	**60**	**64,800**	**15**	**240**	**18**	**10.53**	**104.17**	**55.15**	**5.2**	**9.9**	**1.9**	**1 h 15 min 57 s**	**80.58**	**17,946.75**	**10,953.91**
8	60	79,200	15	240	22	10.53	104.17	45.12	4.3	9.9	2.3	3 h 30 min 3 s	80.60	17,978.32	11,092.49

* *Highlighted (Bold) results corresponding to the most appropriate cocowood FE model for this study*.

A Python program code was used to integrate the typical coconut stem form, complex structure, material properties, and loads acting on the model. The program code automatically reproduced the palm stem model, which in turn was exported to Strand7 to perform the analyses, and the relevant results were retrieved again by the Python program for easy of data evaluation and presentation. The code also considered the progressive reduction of the stem projected area due to higher deflections experienced as the wind speed increased up to 23 m/s.

No damping or swaging effects (i.e., dynamic analyses) during extended high wind events were considered herein as a pilot study showed a relative low-frequency response of about 0.2 Hz for the first natural frequency bending mode of vibration; the modal analysis was done around the equilibrium position (i.e., the non-deformed stem position) from the static geometry calculated around the unloaded state. The vortex-shedding effect was also disregarded due to two main reasons: (i) the high bending capacity (i.e., nearly to quarter circle for wind speeds >23 m/s) and the marked streamlining effect [i.e., foliage moves into alignment with the wind direction (flag) as the wind force increases] allowed reducing the palm crown/stem projected areas and the pressure drag. Hence, the whole structure reduced the wind surface, the bending moments and the vortex shedding phenomenon. (ii) The vortex shedding frequency was found to be very low (i.e., about 0.077 Hz) for an average slender coconut stem diameter of 237.8 mm, and the critical wind speed under investigation (i.e., 23 m/s), and thereby the quasi-static state portion of the movement dominated the analyses. Thus, non-linear static analyses (NLA) were performed taking into account the cocowood stem's geometric non-linear effect (GNL) produced by the large deflections in the model.

The finite element model of the coconut stem-tissue was investigated under the influence of the non-uniform distributed wind pressure (*P*_*w*_), the top wind force (*F*_*top*_), and the self-weight (*P*) of the whole structure. The *P*_*w*_profile (in N/m^2^) acting along the cocowood FE model was derived from the Australian Standards AS/NZS 4676 (2000) and AS/NZS 1170.2 (2011), and calculated as,

(5) $$P_{w}=(0.5\rho_{air}){\left(f_{h}W_{s}\right)}^{2}\times 1.2$$

where ρ_*air*_ is the density of air equal to 1.225 kg/m^3^ (i.e., at sea level and 15°C), *f*_*h*_ is a coefficient that varies from 0.99 to 1.22 depending on the stem height (i.e., from 0 to 25 m) at which *P*_*w*_ is applied, and *W*_*s*_ is the wind speed in m/s.

Even though wind exerts a distributed non-uniform pressure all over the tree stem together with its crown (i.e., the tree's projected area), the bulk of the wind pressure acts primarily on the center of the crown (Niklas, 1992) and, therefore, a top wind force *F*_*top*_, also known as “drag force” (Mayhead, 1973; Rudnicki et al., 2004; Vollsinger et al., 2005) was calculated (in N) as,

(6) $$F_{top}=P_{w}(25m)\times A_{proj}\times C_{D}$$

where *P*_*w*_ in N/m^2^ was considered at the palm stem top (25 m in this analysis), *A*_*proj*_ (in m^2^) is the projected area by the coconut leaves at the palm stem's crown, and *C*_*D*_ is the drag coefficient assumed to be constant and equal to 1. The coconut palm projected area *A*_*proj*_, equal to 1.2 m^2^ in this study, was derived from related studies (Rich et al., 1995; Rudnicki et al., 2004; Vollsinger et al., 2005). The vertical self-weight load (*P*) was calculated in terms of gravity and mass of the cocowood structure.

### Finite element analyses

To fulfil the purposes in this study, a total of 11 finite element analyses (FEA) were carried out over the 3D cocowood FE model exposed to the wind speed of 23 m/s (i.e., Gale tornado, according to the Fujita tornado scale; Cullen, 2002); the wind speed that was determined in Gonzalez (2015) as the critical wind speed (i.e., the wind at which the material tissue starts reaching stress at failure) under the equivalent bending moment of 64.4 kN.m.

The analyses were performed by varying the characteristic cocowood average maxima fibrovascular bundle degree of orientation θ_*max*_ from 0° to 51°. It thus changed the characteristic cocowood fibrovascular tissue system in terms of θ, given by Equation (2). Higher θ_*max*_ variations were not examined as the results from the above-mentioned range showed well-defined trends for both the material stress at failure and the palm bending stiffness. Furthermore, the optimal average maxima fibrovascular bundle degree of orientation was determined by the point at which the cocowood tissue endured high load without considerably reducing its compressive strength and bending stiffness.

The bending stiffness is defined herein as the force required to produce a unit deflection of the coconut palm stem. The palm bending stiffness *k* was calculated (in kN/m) as (Ugural, 2008),

(7) $$k=\frac{F_{th}}{\delta_{\text{h}}\;}$$

where *F*_*th*_ (in kN) is the horizontal reaction force acquired from the FEA in Strand7, and δ_*h*_ (in m) is the maximum horizontal displacement in the wind direction at the top/middle point of the cocowood model.

Due to the orthotropic nature of the coconut stem-tissue, the Tsai-Hill failure criterion (Jones, 1999; Rao et al., 2008), which is a variation of the von Mises criterion (Hull and Clyne, 1996) and the Tsai-Wu criterion (Cabrero and Gebremedhin, 2010), was used to predict and evaluate the circumstances under which stress at failure was likely to occur (i.e., the material damage when the stresses produced by the progressive loading conditions rose beyond the material strength). Specifically, the material failure was predicted by this method when the failure index (*FI*) was ≥1. The referred failure index (*FI*) was calculated as (Jones, 1999; Rao et al., 2008),

(8) $$\begin{array}{l} FI={\left(\frac{\sigma_{L}}{MOR_{L}}\right)}^{2}+{\left(\frac{\sigma_{R}}{MOR_{R}}\right)}^{2}+{\left(\frac{\sigma_{T}}{MOR_{T}}\right)}^{2}\\ \;-\sigma_{L}\sigma_{R}\left[\frac{1}{MOR_{L}^{2}}+\frac{1}{MOR_{R}^{2}}-\frac{1}{MOR_{T}^{2}}\right]\\ \;-\sigma_{L}\sigma_{T}\left[\frac{1}{MOR_{L}^{2}}-\frac{1}{MOR_{R}^{2}}+\frac{1}{MOR_{T}^{2}}\right]\\ \;-\sigma_{R}\sigma_{T}\left[\frac{-1}{MOR_{L}^{2}}+\frac{1}{MOR_{R}^{2}}+\frac{1}{MOR_{T}^{2}}\right]\\ \;+{\left(\frac{\tau_{LR}}{\tau_{max_{LR}}}\right)}^{2}+{\left(\frac{\tau_{RT}}{\tau_{max_{RT}}}\right)}^{2}+{\left(\frac{\tau_{TL}}{\tau_{max_{TL}}}\right)}^{2} \end{array}$$

where σ_*L*_, σ_*R*_, and σ_*T*_ (in MPa) are stresses in the *L, R*, and *T* directions, respectively. *MOR*_*L*_*, MOR*_*R*_, and *MOR*_*T*_ (in MPa) are the compressive strengths in the *L, R*, and *T* directions, respectively. τ_*LR*_, τ_*RT*_, and τ_*TL*_ (in MPa) are shear stresses in the longitudinal-radial (*LR*), radial-tangential (*RT*), and tangential-longitudinal (*TL*) planes, respectively. τ_*maxLR*_, τ_*maxRT*_, and τ_*maxTL*_ (in MPa) are the material shear strengths in the *LR, RT*, and *TL* planes, respectively.

## Results

### Tangential fibrovascular bundle degree of orientation

The results for this part of the study are based on 26,400 fibrovascular bundle degree of orientation measurements corresponding to 264 cocowood discs. Preliminary analysis showed no significant differences in the acquired global measurements between the different coconut growing sites (i.e., Fiji and Samoa). Thus, the results in this section represent the analysis of the coconut palms sourced from Fiji and Samoa islands altogether.

**Figure 7:**
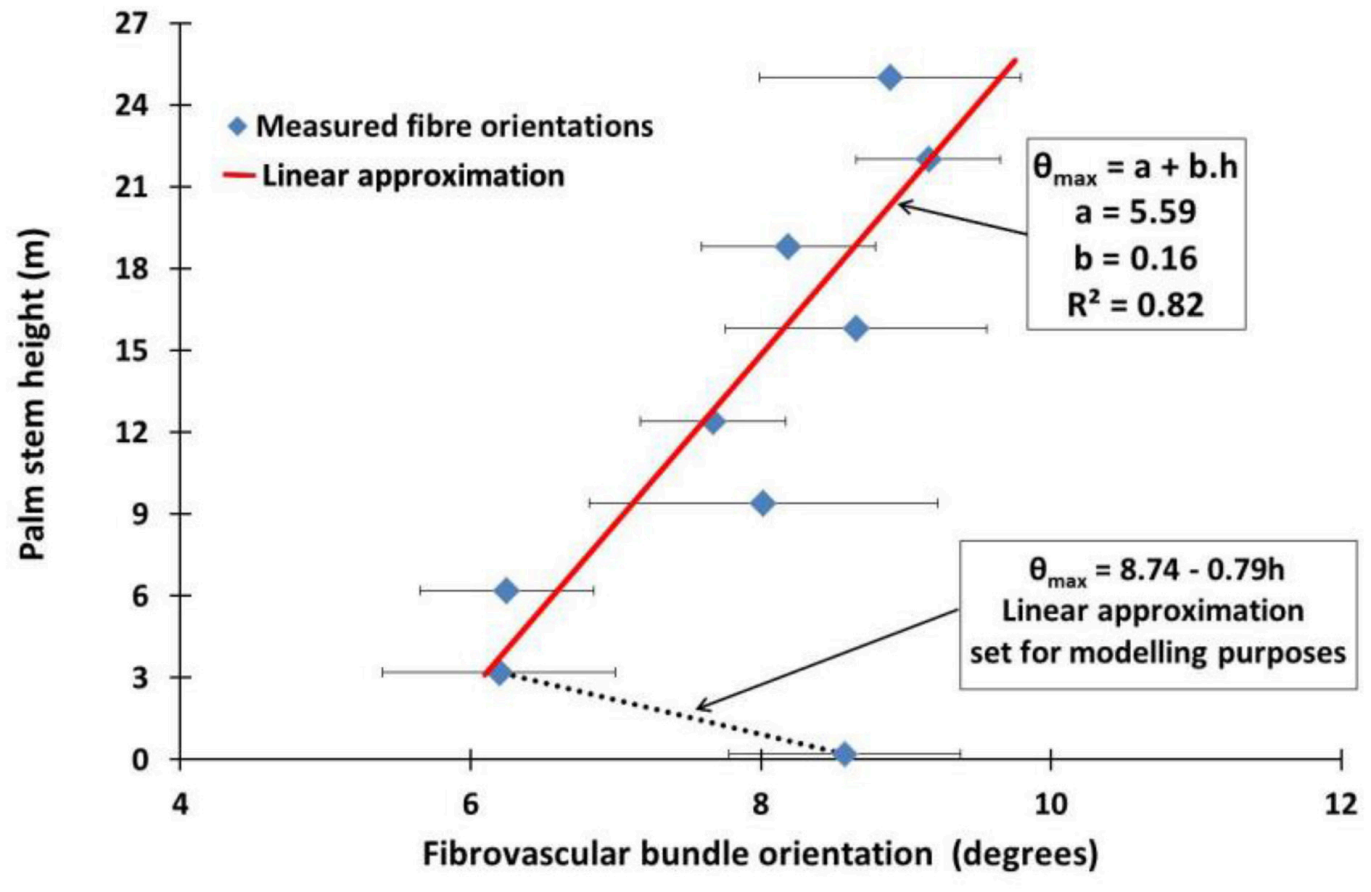
**The average maxima fibrovascular bundle degree of orientation θ_*max*_ against the palm stem height is plotted in the figure**. Two specific patterns can be observed. First, the average maxima fibrovascular bundle degree of orientation decreases with the stem height from 0 to 3.2 m and second, quasi-linearly increases with the stem height above 3.2 m. Thus, the highest deviation of fibrovascular bundles occurs toward the upper part of the palm stem where the higher flexibility of the material is needed during extreme loading conditions.

Table 4 gives the average values of the local maximum and minimum fibrovascular bundle degree of orientations, as the average maxima fibrovascular bundle degree of orientation θ_*max*_ at all investigated stem heights. The average maxima fibrovascular bundle degree of orientation θ_*max*_ is found to vary with the palm stem height and range from a minimum degree of 6.20° at 3.2 m high to a maximum value of 9.15° at 22 m of stem height. θ_*max*_ is found to be 8.58° at the bottom of the coconut palm stem (see Figure 7). Based on these observations and, as no clear correlation can be found in Figure 7 between the measurement at the ground elevation and all subsequent measurements, two linear equations are chosen in this study to characterize the average maxima fibrovascular bundle degree of orientation θ_*max*_ for senile coconut stem tissues. Note that the linear approximation that characterizes the tangential degree of orientation up to the stem height of 3.2 m, was set only for modeling and discussion purposes. θ_*max*_ (in degrees) is then expressed as function of the palm stem height *h* (in m) as,

(9) $$\{\begin{array}{l} \theta_{max}\left(h\right)=8.74-0.79\times h\text{ for }h\leq 3.2\text{ m}\\ \theta_{max}\left(h\right)=5.59+0.16\times h\text{ for }h>3.2\text{ m} \end{array}$$

**Table 4:** **Average maxima tangential fibrovascular bundle degree of orientation (θ_*max*_) for the studied coconut palms**.

**Stem height (m)**	**Local *max/min***		**θ*max***	
	**Average *max* (deg)**	**Average *min* (deg)**	**Average (deg)**	***CoV***
0.2	7.14	−10.02	8.58	0.37
3.2	5.63	−6.77	6.20	0.39
6.2	4.84	−7.65	6.25	0.35
9.4	5.97	−10.07	8.02	0.29
12.4	6.45	−8.89	7.67	0.29
15.8	6.92	−10.38	8.65	0.34
18.8	7.07	−9.31	8.19	0.38
22.0	7.01	−11.30	9.15	0.35
25.0	8.75	−9.04	8.89	0.40

**Figure 8:**
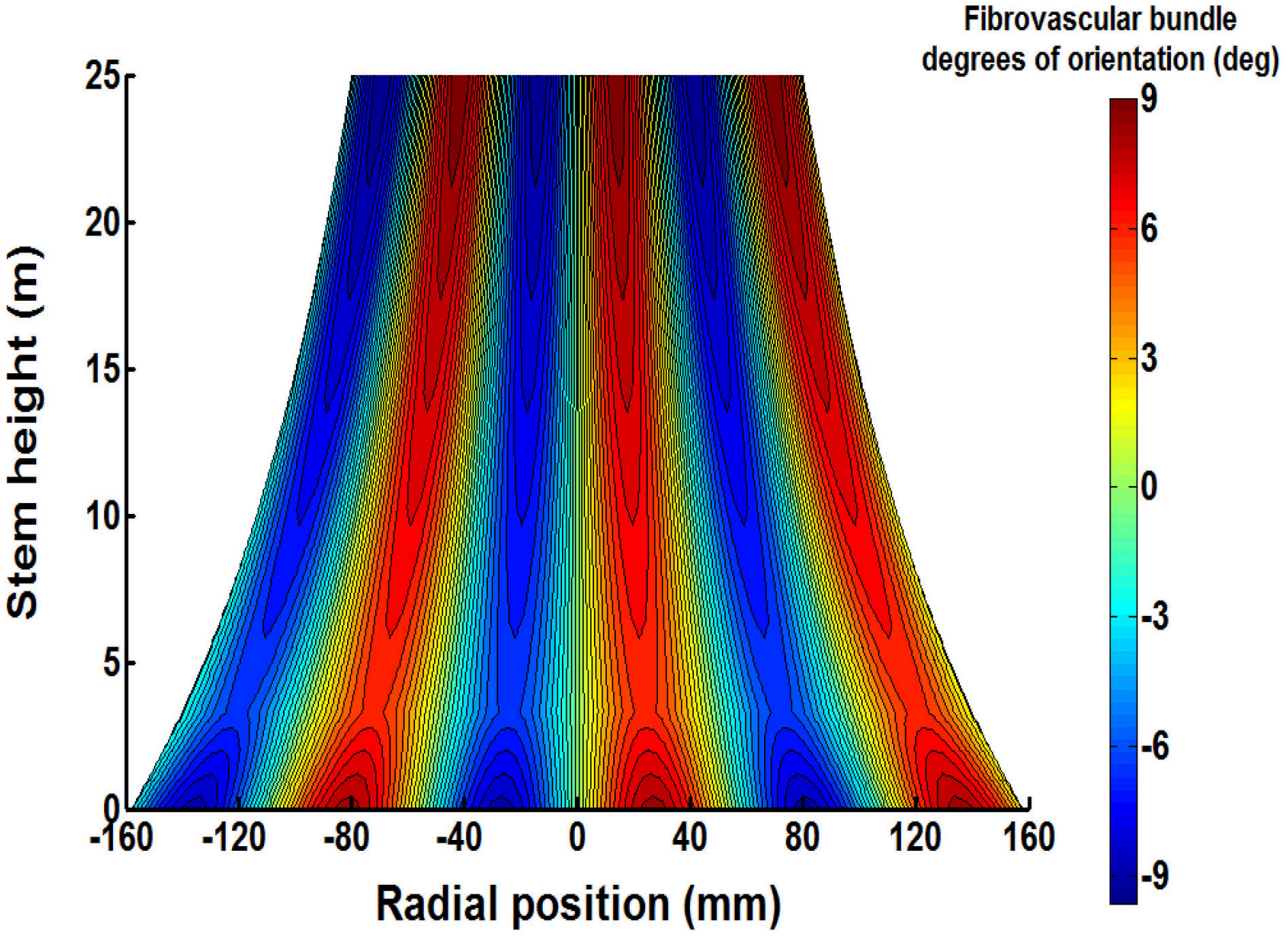
**The proposed typical triple helix distribution of tangential fibrovascular bundle degrees of orientation is shown on the plane of symmetry of a characteristic senile coconut palm 25 m stem height**. The cocowood structure in the figure shows an apparent pattern of fibrovascular bundles with a maximum deviation of 9.15°. Note also a different fibrovascular bundle configuration up to a stem height of 3.2 m.

Table 5 gives the average values of natural frequencies found at the investigated stem heights. No clear correlation between the natural frequency of the tangential fibrovascular bundle degree of orientation and the palm stem height can be found and, therefore, a unique natural frequency *f* of 1.45 mm/mm is considered herein for the entire palm. Thus, the proposed tangential fibrovascular bundle degree of orientation presented in Figure 4 was calculated by using Equation (2) with inputs from Equation (3), Equation (9), and the above mentioned natural frequency *f*. Similarly, Figure 8 maps the proposed tangential fibrovascular bundle degree of orientation for the whole structure of a characteristic coconut stem of 25 m in height.

**Table 5:** **Average values of natural frequencies at all investigated heights and the representative natural frequency *f* for all the studied cocowood discs**.

**Stem height (m)**	***f* (mm/mm)**	***CoV***	**Total number of discs**
0.2	1.49	0.208	34
3.2	1.41	0.202	28
6.2	1.53	0.159	38
9.4	1.46	0.250	26
12.4	1.53	0.175	40
15.8	1.40	0.185	29
18.8	1.41	0.263	37
22.0	1.42	0.229	15
25.0	1.29	0.267	17
Average (weighed)	**1.45**	0.215	264

*Bold result corresponding to the representative natural frequency of the whole coconut palm stem-tissue*.

### Effects produced by the variation of the tangential fibrovascular bundle degrees of orientation on the cocowood mechanical performance

**Figure 9:**
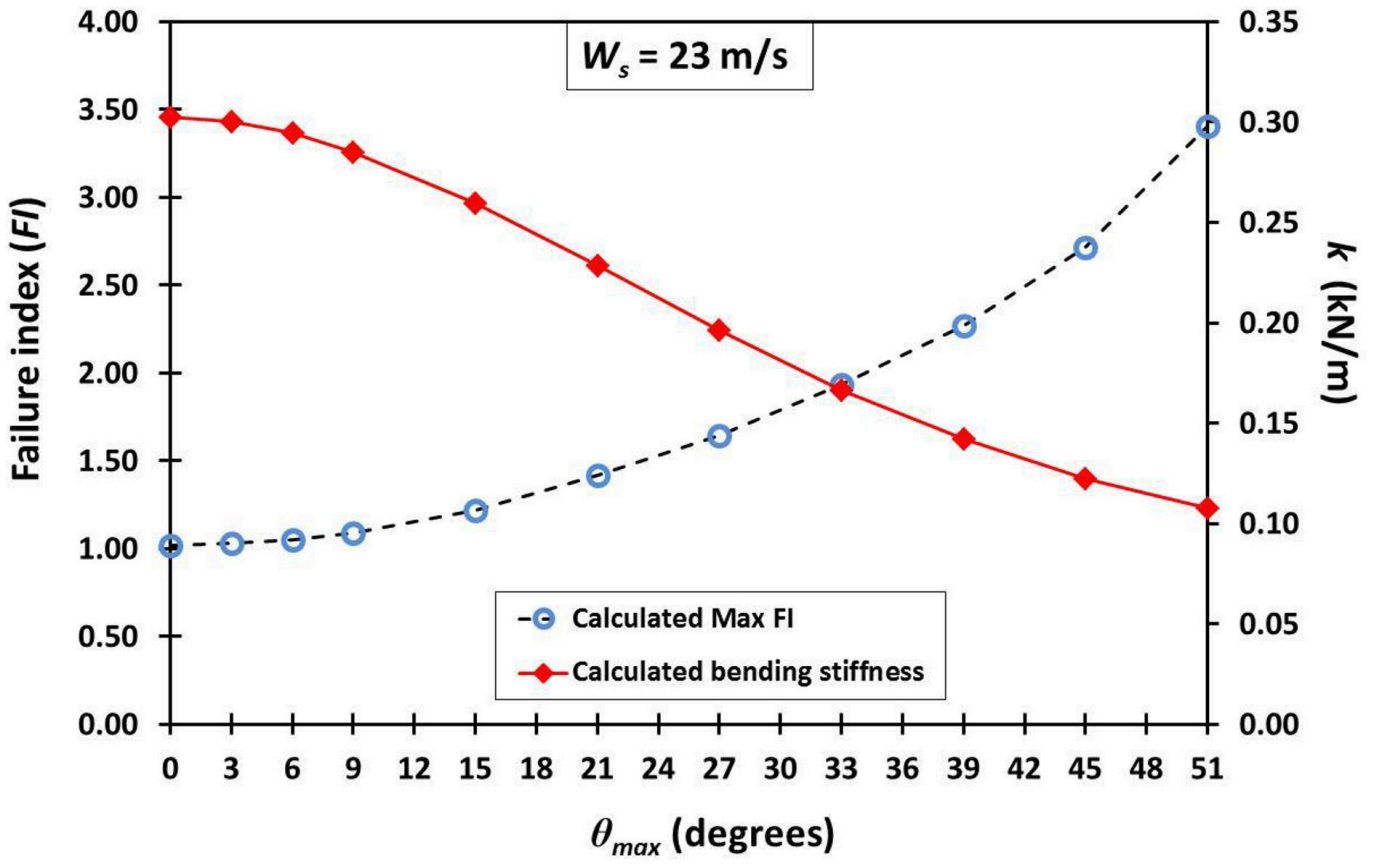
**An insignificant increase in material failure index *FI* (segmented line with blue circles) is observed for fibrovascular bundle degrees of orientation up to 9°**. Beyond this point, the slope of the *FI* curve starts to drastically increase reaching a *FI* of about 3.5 when the angle varied to 51°. For the θ_*max*_ of 15°, there is a sudden *FI* increment that is three times higher than the previous value at 9°. Similarly, the palm bending stiffness *k* (red line with rhombi) is sharply reduced by nearly three times from 9° to 15°. The identified trends become higher and lower, respectively, for the greater values of θ_*max*_.

Table 6 gives the results from the FEA for every variation of the average maxima fibrovascular bundle degrees of orientation (0°–51°) when the wind speed of 23 m/s is acting on the whole cocowood structure. Figure 9 shows both the palm stiffness *k* and the maximum failure stress *FI*, plotted against the variation of average maxima fibrovascular bundle degrees of orientation (0°–51°) for the analyzed wind speed. In the main, the research findings show a significant increase in material failure index and a severe decrease of the palm bending stiffness for fibrovascular bundle degrees of orientation >9°. It can also be noticed that both factors (i.e., *FI* and *k*) are critically reduced when the average maxima fibrovascular bundle degrees of orientation become ≥33°.

**Table 6:** **FEA results for the variation of θ_*max*_ at the critical wind speed of 23 *m/s***.

**FEA no**.	***θmax* (deg)**	**Max *FI***	***FI* increment (%)**	***F_th_* (kN)**	***δ_h_* (m)**	***k*(kN/m)**	***k* drop (%)**
1	0	1.021		3.036	10.046	0.302	
2	3	1.029	0.76	3.033	10.101	0.300	0.66
3	6	1.052	2.21	3.021	10.262	0.294	1.94
4	9	1.091	3.60	3.002	10.526	0.285	3.12
5	15	1.219	10.53	2.941	11.333	0.260	9.01
6	21	1.416	13.90	2.844	12.450	0.228	11.98
7	27	1.646	13.96	2.689	13.687	0.196	13.98
8	33	1.931	14.76	2.518	15.109	0.167	15.20
9	39	2.268	14.87	2.374	16.689	0.142	14.64
10	45	2.718	16.54	2.145	17.531	0.122	13.96
11	51	3.406	20.20	1.954	18.137	0.108	11.97

## Discussion

Similar to some monocotyledonous palms (e.g., *W. robusta* palms), senile coconut palms are tall and slender with an aspect ratio (about 80) that tends to indicate excellent stability (Slodicak and Novak, 2006) with a high bending capacity during strong wind conditions (Fratzl and Weinkamer, 2007). Indeed, from the acquired results it has been revealed a cocowood structure that is, from the structural mechanics point of view, stronger toward the palm stem's bottom-periphery, where the greater concentration of fibrovascular bundles were found. Interestingly, the cocowood structure at the stem's top part close to the leaves has the minimum diameter and is the least dense part of the stem with the highest deviation of fibrovascular bundles, which structurally means it is the most flexible part of the whole structure. This strategy may likely permit the palm stem to better bend or efface itself under high wind conditions with the leaves streamline effect offering a very low resistance to wind pressure. Yet, it is a hypothesis that might be proved by future studies.

The calculated average maxima fibrovascular bundle degree of orientation at the bottom part of the stem (see Figure 7) markedly differs from those for the higher elevations. It clearly reflects a different configuration of the cocowood fibrovascular tissue system at this section of the palm stem. This occurs due to the transition between the stem-tissue and root systems, both having structurally and functionally different configurations (Niklas, 1992). For modeling purposes, these properties have been represented in this study by simple linear equations that match the dissimilar property regions. However, the atypical pattern found close to the bottom of the palm stem needs further investigation, likely from a different approach (e.g., microscopic), in order to accurately depict the structure at this region of the coconut stem.

Note that the average maxima fibrovascular bundle degree of orientation θ_*max*_ of 8.58° was found in this study at the bottom of the characteristic senile coconut palm stem whereas the θ_*max*_ of 12° was reported by Kuo-Huang et al. (2004) for juvenile palms (6 m high) at the same stem location. It can be inferred that tangential fibrovascular bundles likely become less deviated in senile cocowood due to the structural adaptation process experienced as coconut palm stems grow taller and have to resist higher external loading conditions; a common phenomenon in plant species (Zhang et al., 2012).

The cocowood mechanical response observed when varying the degrees of orientation of fibrovascular bundles has in principle (i.e., the greater the fiber orientation, the inferior the tree stem mechanical response, and vice versa) some similarity to that of hardwoods and softwoods. For example, the woody stem spiral configuration of the southern Utah's Ponderosa pine (*Pinus ponderosa*) species was studied by Leelavanichkul and Cherkaev (2004) to determine the influence of fiber orientations (varying from 21.2° to 90°) on the trunk strength under three progressive wind forces expressed in terms of bending moments (2.26, 22.59, and 90.39 kN·m). The results showed that the Ponderosa pine trunk failed when the critical fiber angle was set at 37°. The critical average maxima fibrovascular bundle degree of orientation of 33° was found herein. The reason of such similar findings may have its foundations on (i) the similar fibrovascular-cellular structure (i.e., an assembly of prismatic or polyhedral cells with solid edges and faces packed together to fill space; Gibson, 2005) that both cellular solids (i.e., woody plants and palmwoods) have in common, and (ii) the related mechanical characteristics that are mutual for tree stems in order to support static and dynamic loads, store and release elastic energy, bend through large angles, and resist buckling and fracture.

When the fibrovascular bundle degrees of orientation varied between 0° and 9°, a superior cocowood biomechanical response was identified in terms of the stem bending stiffness and the material capacity to undergo failure due to generated stresses and strengths. Average maxima fibrovascular bundle degrees of orientation beyond this limit (θ_*max*_ of 9°) significantly reduced the palm bending stiffness and weakened its compressive strength with a consequent increase in material failure (*FI*). Thus, a θ_*max*_ = 9° is identified as the *maximal* fibrovascular bundle degree of orientation, defined in Leelavanichkul and Cherkaev (2004) as the maximum fiber angle that does not substantially reduce/weaken the stiffness and strength of a tree. It can thus be concluded that senile cocowood fibrovascular tissue systems are optimally arranged complex structures, partially because of the typical variation of average maxima fibrovascular bundle degrees of orientation (i.e., 6.2° to 9.1°) that was found herein to be within the limits of the *maximal* fibrovascular bundle degree of orientation defined above. Where nature has already optimized the structure of a living organism, it is assumed there must be a reason for it. In fact, what has been observed in the current analysis is not just a random causality of the biomechanical behavior produced by the cocowood fibrovascular tissue system; on the contrary, it is without doubt, a phenomenon that reflects an optimized cocowood fibrovascular development resulting from the natural adaptation of the material to resist varying conditions of external forces [e.g., gravity (biomass), wind, and rain water], during the course of millions of years of evolution. Wind forces, for example, become critical as trees age and grow taller, particularly because, during high wind episodes, the forces induced on the stems, foliage, and roots can reach critical bounds. If surpassed, these forces will result in failure. Further, while strong winds may occur only a few times in a tree's life, its resistance capability to overturning or breaking during these extreme weather conditions is critical to its survival (Vogel, 1989, 1996).

The acquired results in this study evidenced an efficient cocowood fibrovascular tissue system that highlighted its optimal local design in terms of the tangential deviation of the axial fibrovascular bundles. The present analyses significantly advanced the understanding of the cocowood fibrovascular tissue system and, concomitantly, of the monocotyledonous palms. The research findings have a significant future potential for innovative material/structure concepts; e.g., the representative cocowood fibrovascular bundle degrees of orientation proposed herein may serve as a biomimetic inspiration to produce superior engineered wood products (EWPs) such as coconut fiber reinforced polyethylene composites (Brahmakumar et al., 2005) and/or spirally wood-laminated composite poles (Piao et al., 2008; Berard et al., 2011). Yet, further research from a multidisciplinary approach should be carried out to reveal other properties and underlying principles of the biomaterial at the macroscopic, microscopic, ultrastructural, and biochemical levels of hierarchical structure. Such studies would also improve our understanding of the cocowood fibrovascular development; a system that has evolved in accordance with engineering principles long before these principles were known.

## Author contributions

Dr. OG performed the measurements and analyses, derived the equations, drew the figures, and wrote the paper. Dr. KN supervised the work, reviewed the analyses, and wrote the paper.

### Conflict of interest statement

The authors declare that the research was conducted in the absence of any commercial or financial relationships that could be construed as a potential conflict of interest.
